# Spatially confined hydration for robust underwater adhesion

**DOI:** 10.1126/sciadv.aea3097

**Published:** 2025-11-05

**Authors:** Gang Lu, Rui Ma, Jian Lu, Yuanhao Chang, Ming Li, Eduardo Saiz

**Affiliations:** ^1^ Yantai Institute of Coastal Zone Research, Chinese Academy of Sciences, Shandong 264003, China.; ^2^ Petroleum Systems Engineering, Faculty of Engineering and Applied Science, University of Regina, Regina, Saskatchewan S4S 0A2, Canada.; ^3^ Centre of Advanced Structural Ceramics, Department of Materials, Imperial College London, London SW7 2AZ, UK.; ^4^ Department of Data and Systems Engineering, The University of Hong Kong, Pok Fu Lam, Hong Kong, China.

## Abstract

Underwater adhesion has long been limited by interfacial water’s paradoxical role as both bonding mediator and failure initiator. We present a confined hydration adhesive tape (CHAT) that harnesses water as a molecular architect through spatial hydration management. By confining water penetration to sub–8-micrometer depths, we create a dynamic interface where hydration-activated hydrogen bonds enable adaptive, high-density interfacial connections, and hydrophobic nanodomains maintain bulk integrity via entropic water exclusion. This orchestrated hydration yields an interfacial toughness of 6 kilojoules per square meter (>1.8× literature benchmarks; 1.4 to 3.8× commercial tapes), while preserving stability across harsh conditions (pH 1 and 13, 3.5% saline). Multiscale experiments and simulations reveal water’s triple role as a hydrogen bond catalyst at the interface, a dynamical reorganizer of supramolecular networks, and a mechanical decoupler of interfacial adhesion/bulk cohesion. By establishing interfacial water as a design variable rather than a compromise, CHAT opens avenues for marine, biomedical, and industrial applications where water-resistant adhesion is critical.

## Teaser

Transforming water from a foe into an architect achieves record-breaking underwater adhesion.

## INTRODUCTION

Adhesive tapes that offer instant and reversible adhesion are indispensable across industries from automotive to biomedicine (*1*–*6*). However, their performance falls catastrophically in aqueous environments because interfacial water prevents direct contact and compromises binding interactions, leading to delamination (*7*–*10*).

The pursuit of effective underwater adhesion has historically followed two primary strategies, inspired by different natural organisms. The first, inspired by the gecko and octopus, focuses on surface engineering (*11*, *12*). This includes creating micro- and nanoscale fibrillar structures (e.g., pillars and suckers) to displace water via capillary action, increase friction, and achieve mechanical interlocking (*13*, *14*). While effective for certain applications, these approaches often lack robust chemical bonding and can be challenging to apply to complex or soft surfaces. The second strategy, inspired by mussels and barnacles, focuses on molecular engineering to manage water and form strong bonds at the interface (*3*, *15*–*19*). A seminal advance was the development of double-sided tapes (*20*), which use hydrophilic polymers to absorb interfacial water, enabling covalent and noncovalent bonding to diverse surfaces. Alternatively, supramolecular chemistry has been harnessed to create strong underwater bonds (*21*–*24*); for instance, Kim *et al.* developed a supramolecular Velcro based on hydrophobic host-guest interactions (*25*), achieving adhesion strengths up to 1.1 MPa. Moreover, a class of tough and reusable adhesives has been created by leveraging dynamic bonds, such as disulfide linkages or hydrogen-bonding networks (*6*, *9*, *26*).

Despite these notable advancements in adhesive systems (*5*, *27*, *28*), a critical challenge is the inherent trade-off between strength (high-peak stress) and toughness (high-energy dissipation). Strong adhesion necessitates rigid, dense bonds (e.g., covalent or noncovalent) that resist detachment, while tough adhesion requires dynamic, sacrificial bonds (e.g., reversible cross-links) that dissipate energy and blunt crack propagation. This trade-off in water is further exacerbated, as hydration can plasticize the material, weakening both cohesive and adhesive strength. Consequently, achieving a synergy of high strength and high toughness in a fully hydrated state remains a substantial hurdle. To transcend this limitation, we propose a paradigm shift with three integrated design principles: (i) spatial water management, using a hydrophobic matrix to repel bulk water while polar motifs control interfacial hydration; (ii) conformal contact, enabled by a hydration-plasticized surface layer that adapts to substrate topography; and (iii) bulk resilience, provided by an energy-dissipating supramolecular network that maintains mechanical integrity.

Here, we introduce a spatially confined hydration strategy that transforms interfacial water from a foe to a functional element. Through molecularly engineered supramolecular cooperative networks (SCNs)—comprising strong polar motifs, weak dynamic linkers, and a flexible hydrophobic backbone—we develop confined hydration adhesive tapes (CHATs) that spatially orchestrate water distribution. This design creates a dynamic interfacial zone (<8 μm) where hydration-activated hydrogen bonds enable adaptive, high-density connections, while hydrophobic nanodomains shield the bulk from water plasticization, preserving mechanical integrity. This decoupled control allows CHAT to overcome the classic strength-toughness trade-off, achieving >7-MPa shear strength and >6 kJ m^−2^–interfacial toughness on engineering materials, with stability across extreme conditions (pH 1 and 13, 3.5% saline, urea). By programming interfacial water as a design variable—rather than simply excluded—this work establishes a paradigm for next-generation underwater adhesives in marine, biomedical, and industrial applications.

## RESULTS

### Molecular design and characterization

**Fig. 1.**
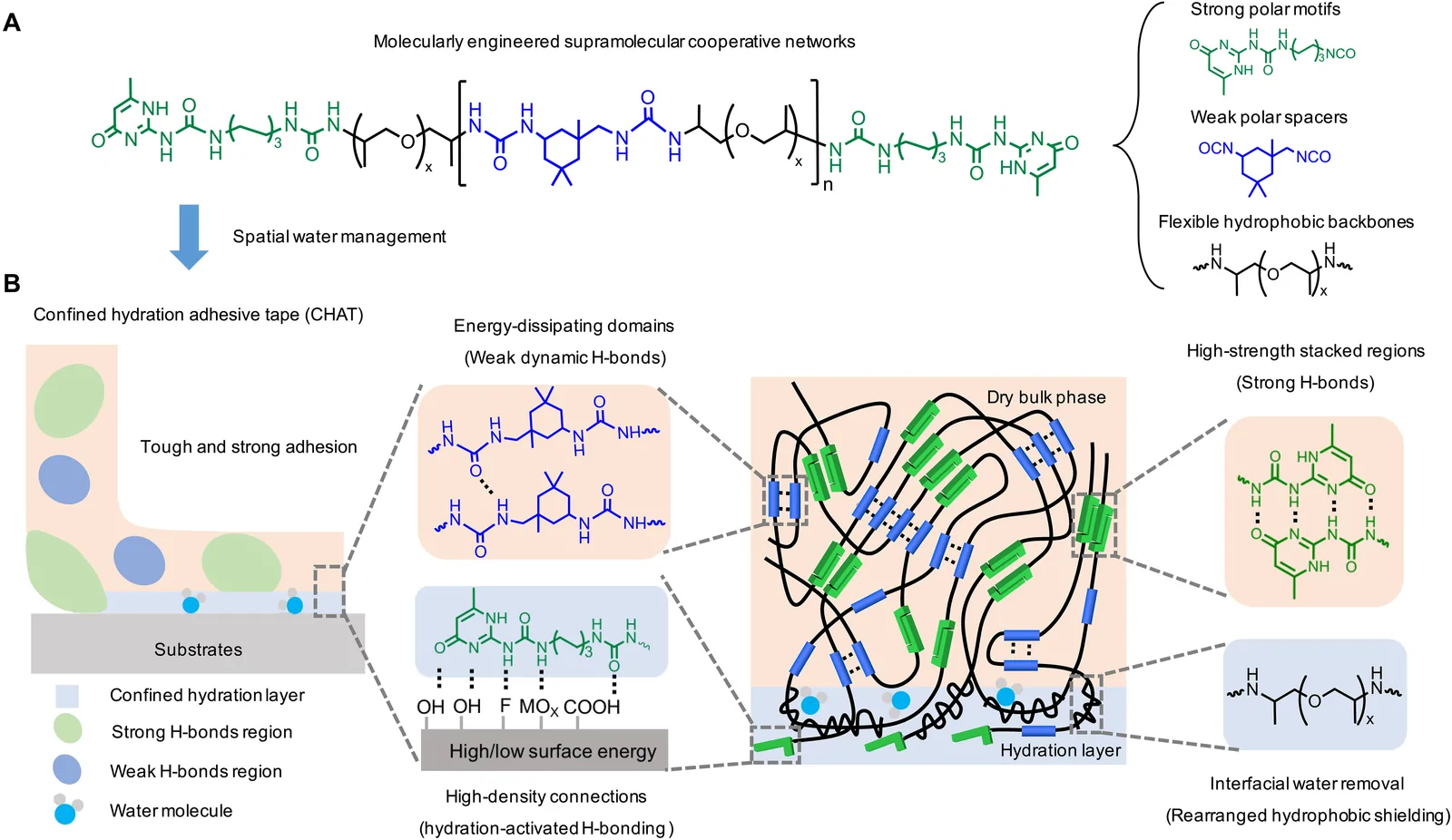
Molecular-scale engineering and precision hydration control of the CHAT. (**A**) Chemical structure of the SCN, integrating three key motifs: (i) strong polar groups from UPy moieties (green) that form self-complementary quadruple hydrogen-bonded dimers and nanostacks for strength; (ii) weak dynamic linkers from IPDI segments (blue) that enable energy dissipation via reversible bond exchange; and (iii) a soft, hydrophobic matrix from flexible polyetheramine chains (OPG-2NH_2_, black) that provides conformability and bulk water resistance. (**B**) The spatially confined hydration mechanism enables robust underwater adhesion. Upon hydration, water penetration is kinetically and thermodynamically confined to a superficial layer (<8 μm). In this hydrated interface, water plasticizes the polymer, enabling conformal contact and activating dynamic H-bond dissociation/reformation to generate a high density of binding sites. Simultaneously, the bulk material remains unplasticized because of restrictive hydrophobic nanodomains, preserving its mechanical toughness and cohesive strength through the energy-dissipating supramolecular networks. This decoupled design—hydration-activated interface and water-resisting bulk—synergistically overcomes the classic strength-toughness trade-off in wet environments.

Our molecular design strategy aims to create a supramolecular system that spatially manages water to achieve both tough and strong underwater adhesion. As illustrated in Fig. 1, we propose a hierarchical architecture integrating three complementary functional motifs: (i) high-strength polar groups. 2-ureido-4-pyrimidinone (UPy) moieties form self-complementary hydrogen-bonded dimers that further assemble into nanoscale stacks (*6*, *29*–*31*). These robust, reversible noncovalent interactions provide mechanical reinforcement and serve as a high-density of binding sites for hydration-activated adhesion. (ii) Energy-dissipating motifs. Weakly polar isophorone diisocyanate (IPDI) segments (blue) introduce dynamic bond exchange and localized mobility. This enhances toughness by dissipating mechanical energy under stress and prevents catastrophic crack propagation. (iii) Hydrophobic soft matrix. Flexible polyetheramine chains [poly (propylene glycol) bis (2-aminopropyl ether), OPG-2NH_2_; M_n_ = 2000 g mol^−1^, black] form a continuous soft and hydrophobic phase. This matrix enables conformal contact with substrates and is crucial for repelling water to confine hydration to a superficial layer, thereby preserving the bulk mechanical properties. This design—combining rigid nanostacks, dissipative domains, and a ductile matrix—is intended to ensure simultaneous bulk toughness, interfacial adaptability, and bulk mechanical resilience (*29*, *32*).

To realize this design, we synthesized a series of SCNs via one-pot polycondensation reaction between OPG-2NH_2_, UPy moieties, and IPDI spacers at varying molar ratios (detailed in table S1). Comprehensive characterization confirms the successful synthesis and formation of the intended nanostructures. The successful incorporation of UPy motifs and formation of strong hydrogen-bonded dimers was verified by proton nuclear magnetic resonance (^1^H NMR) spectra (fig. S1), which showed characteristic N−H proton resonances between 10.0 and 14.0 parts per million (*30*, *33*). Gel permeation chromatography (GPC) (fig. S2) confirmed the formation of oligomers with controlled molecular weights ranging from 2584 to 9022 g mol^−1^ and low polydispersity of 1.2. Fourier transform infrared (FTIR) spectroscopy confirmed the completion of the reaction, as evidenced by the absence of the isocyanate peak at ~2265 cm^−1^ (fig. S3).

The resultant oligomers were hot-pressed into homogeneous, free-standing films, termed dry adhesive tapes (DATs). The microstructure of the DATs was probed to confirm nanodomain formation. X-ray diffraction (XRD) profiles (fig. S4) showed that DAT-0 (highest UPy content) was semicrystalline, with sharp peaks indicating nanosized stacks of UPy dimers. The crystallinity was progressively disrupted with increasing flexible chain content (DAT 1-3), resulting in more amorphous states. Differential scanning calorimetry (DSC) (fig. S5) further confirmed this morphology, showing a distinct endothermic melting peak around 150°C for DAT-0 attributed to UPy nanocrystals, and glass transition temperatures ranging from −10° to −25°C, indicating enhanced segmental flexibility in DATs 1-3. Critically, small-angle x-ray scattering (SAXS) analysis revealed a primary scattering peak at *q* = 0.445 nm^−1^, corresponding to a *d*-spacing of 14.1 nm (fig. S6). This confirms the existence of periodic nanodomain segregation, consistent with the intended hydrophobic/hydrophilic phase separation driven by the aggregation of UPy stacks and the hydrophobic OPG chains. Guinier analysis at low-*q* yielded a radius of gyration (*R*_g_) of 6.7 nm for the UPy-rich clusters (fig. S7). Collectively, these results validate our molecular design, confirming the successful creation of SCNs with the engineered hierarchical structure.

### Tough and strong underwater adhesion

**Fig. 2.**
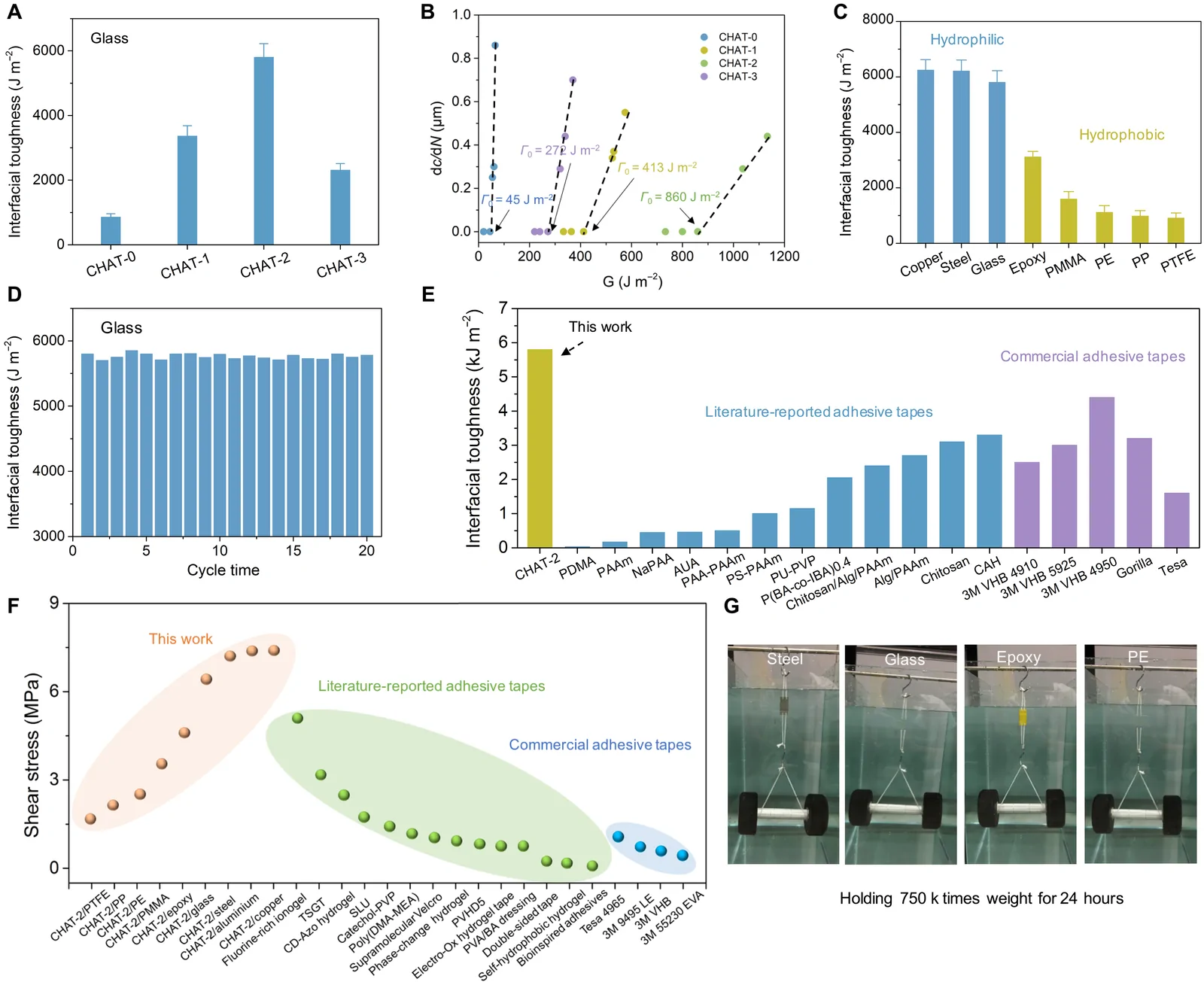
Tough and strong underwater adhesion. (**A**) Interfacial toughness of CHAT samples on glass substrates via a 90° peeling test at 50 mm min^−1^. (**B**) Interfacial fatigue threshold of CHAT samples on the glass substrate via a standard 90° peeling tests under cyclic loading conditions. (**C**) Interfacial toughness of CHAT-2 on the hydrophilic and hydrophobic substrates. (**D**) Interfacial toughness of CHAT-2 on the glass substrate after 20 cycles of loading. (**E**) Benchmarking the interfacial toughness of CHAT-2 against literature-reported and commercial adhesive tapes. (**F**) Benchmarking the lap shear strength against the literature-reported and commercial adhesive tapes. (**G**) Underwater demonstrations of CHAT-2 on diverse substrates, e.g., steel, glass, epoxy, and PE. The bonded samples have an adhesion area of width 20 mm and length 10 mm and a thickness of 50 μm. Data in [(A) and (C)] are means ± SD, *n* = 3.

The underwater adhesion performance of our tapes was evaluated by preparing CHAT through immersion of DAT in water for specified durations. The adhesion testing protocol consisted of first soaking the tape, then pressing it onto a glass substrate with a preload of 10 kPa for 20 s to form the bond, and finally performing a 90° peeling test at 50 mm min^−1^ on the submerged, bonded sample. We found that CHAT-2 exhibited the highest interfacial toughness among all tested samples (Fig. 2A and figs. S8 and S9). CHAT samples consistently showed interfacial toughness two to five times greater than their dry (DAT) counterparts, with CHAT-2 achieving 464% the toughness of DAT-2. To elucidate why CHAT-2 showed superior adhesion performance over other samples, we quantified interfacial fatigue threshold via a standard 90° peeling test under cyclic loading conditions (*6*, *34*, *35*). This critical parameter quantifies the minimum energy to initiate crack growth under cyclic loading (after N cycles), while interfacial toughness measures the energy required to fully separate an interface under quasi-static loading (single cycle). To characterize the interfacial fatigue resistance, we performed cyclic peel tests by applying a periodic peeling force with amplitude *F_a_* for N loading cycles while monitoring the interfacial crack propagation distance *c* as a function of cycle number. The crack growth kinetics were analyzed by plotting the crack extension rate (*dc/dN*) against the applied energy release rate (*G*). The interfacial fatigue threshold (Γ_0_) was determined through linear extrapolation to the *G* axis. As shown in Fig. 2B, the interfacial fatigue threshold of CHAT-2 is 860 J m^−2^, surpassing CHAT-0 (45 J m^−2^), CHAT-1 (413 J m^−2^), and CHAT-3 (272 J m^−2^). Also, the interfacial fatigue threshold of CHAT-2 is 15-fold improvement over that of DAT-2 (fig. S10), which confirms the critical role of spatially confined hydration in creating a stable gradient: a hydrated, dynamic interface supported by a water-resistant bulk. At the crack tip, this allows for continuous breaking and reformation of bonds, which is the mechanism that resists crack propagation. Crucially, the hydrophobic nanodomains in the bulk act as a barrier, preventing further water ingress that would lead to excessive plasticization, swelling, and loss of mechanical integrity.

We systematically investigated the impact of key parameters, e.g., soaking time, preload, and pressing duration, on interfacial toughness through 90° peeling tests at a constant speed of 50 mm min^−1^. The interfacial toughness of CHAT-2 on glass increased with immersion time, reaching approximately 6000 J m^−2^ after 12 hours and plateauing thereafter up to 24 hours (fig. S11A). After a 24-hour soak, the toughness increased notably as the preload was raised from 1 to 10 kPa (with a 20-s press), showing only minor improvements at higher preloads (fig. S11B). With a 24-hour soak and a 10-kPa preload, extending the pressing time to 20 s rapidly improved the interfacial toughness, which stabilized after 100 s (fig. S11C). As shown in Fig. 2C, with 24-hour water soaking followed by a preload of 10 kPa for 20 s, CHAT-2 exhibited remarkable interfacial toughness on various substrates including hydrophilic materials like copper (6240 ± 382 J m^−2^), steel (6210 ± 396 J m^−2^), and glass (5800 ± 420 J m^−2^), as well as on hydrophobic substrates such as epoxy (3110 ± 203 J m^−2^), poly(methyl methacrylate) (PMMA) (1593 ± 273 J m^−2^), polyethylene (PE) (1118 ± 237 J m^−2^), polypropylene (PP) (980 ± 198 J m^−2^), and polytetrafluoroethylene (PTFE) (910 ± 184 J m^−2^). We also evaluated the adhesion ability of CHAT-2 samples that were prepared by immersion under diverse aqueous solutions including phosphate-buffered saline (PBS), acidic (pH 1), basic (pH 13), saline (3.5 wt% NaCl), and urea (5 mg/ml) (fig. S12). After 24-hour immersion and a standard bonding protocol (10-kPa preload for 20 s on glass), CHAT-2 retained its high interfacial toughness, with no notable deviation from the performance achieved in pure water. Further, we measured the interfacial toughness of CHAT-2 bonded on the glass surface with 30-day immersion in acidic (pH 1) or alkaline (pH 13) buffers, tested at various time points (fig. S13). The values showed negligible changes in comparison to those before immersion. These results confirm the robust supramolecular networks and the confined hydration mechanism, enabling universal and durable adhesion in complex, chemically harsh scenarios. Furthermore, the high interfacial toughness of approximately 6000 J m^−2^ on glass substrates was maintained over 20 loading cycles (Fig. 2D), indicating a repeatable adhesion capacity of CHAT. As indicated in Fig. 2E and detailed in table S2, the interfacial toughness of CHAT-2 far exceeds that of both the literature-reported (0.03 to 3.3 kJ m^−2^) (*22*, *24*, *28*, *29*, *36*–*39*) and commercial underwater adhesive tapes (1.6 to 4.4 kJ m^−2^), highlighting the critical role of spatially confined hydration in achieving robust interfacial adhesion.

We further evaluated the lap shear strength of CHAT samples on varying substrates (Fig. 2F). CHAT-2 displayed outstanding underwater shear strength on hydrophilic substrates, including copper (7.5 ± 0.4 MPa), aluminum (7.4 ± 0.3 MPa), steel (7.3 ± 0.3 MPa), and glass (6.5 ± 0.4 MPa), as well as on hydrophobic substrates like epoxy (4.7 ± 0.2 MPa), PMMA (3.5 ± 0.2 MPa), PE (2.6 ± 0.3 MPa), PP (2.2 ± 0.2 MPa), and PTFE (1.7 ± 0.2 MPa). This performance starkly contrasts with that of DAT-2, which ranged only from 0.2 to 1.5 MPa when bonded to the corresponding substrates (fig. S14), which indicates the superior efficacy of CHAT-2. Further, the lap shear strength of CHAT samples was found to be 3.13 to 4.86 times greater than that of DAT samples when sandwiched between glass slides with a preload of 10 kPa for 20 s (fig. S15), highlighting the critical role of spatially confined hydration in achieving strong underwater adhesion. Notably, CHAT-2’s shear strength exceeded those reported in the literature (*15*, *19*–*21*, *25*, *27*, *40*–*47*) and from commercial adhesive tapes when bonded to substrates with distinct surface energies, such as glass, metal, PE, PP, and PTFE (Fig. 2F and table S3). As a demonstration, CHAT-2 is capable of lifting 750,000 times its own weight for at least 24 hours underwater (Fig. 2G), showing its effectiveness on various substrates including steel, glass, epoxy, and PE. In addition, the reproducibility of CHAT-2’s adhesion was also assessed through a cycle of bonding and debonding. Following each cycle, the samples were restored to their original condition using the initial preparation method and retested. The lap shear strength showed no substantial reduction even after 20 bonding and debonding cycles (fig. S16), confirming the adhesive’s repeatability and reusability. Similarly, we evaluated shear stress of CHAT-2 samples that were prepared under harsh conditions. After immersion for 24 hours in various aqueous solutions including PBS (pH 7), urea (5 mg/ml), acidic (pH 1), basic (pH 13), and saline (3.5 wt % NaCl) followed by a standard bonding protocol (10-kPa preload for 20 s), the samples bonded to glass slides demonstrated shear strengths equivalent to those soaked in pure water (fig. S17), showing CHAT’s adhesion tolerance and universality under harsh conditions. To confirm long-term adhesion durability, bonded samples were immersed in saline solution and periodically subjected to lap shear strength testing over 30 days (fig. S18). The bonded glass substrates exhibited no marked degradation in shear strength throughout the immersion period, demonstrating CHAT’s exceptional stability under saline conditions. This outstanding durability positions CHAT as a promising candidate for demanding ocean engineering applications, including underwater equipment repair, offshore energy infrastructure anchoring, and deep-sea resource extraction systems. We also found that molecular weight is a critical design parameter for underwater adhesion performance (Fig. 2A and figs. S9 and S15). It dictates the balance between the density of strong binding motifs and the flexibility of the polymer backbone. An intermediate molecular weight (exemplified by CHAT-2) provides the optimal synergy between these properties, enabling both supreme conformal contact and a high density of hydration-activated bonds, which is essential for achieving record-high underwater adhesion.

### Experimental evidence for confined hydration

**Fig. 3.**
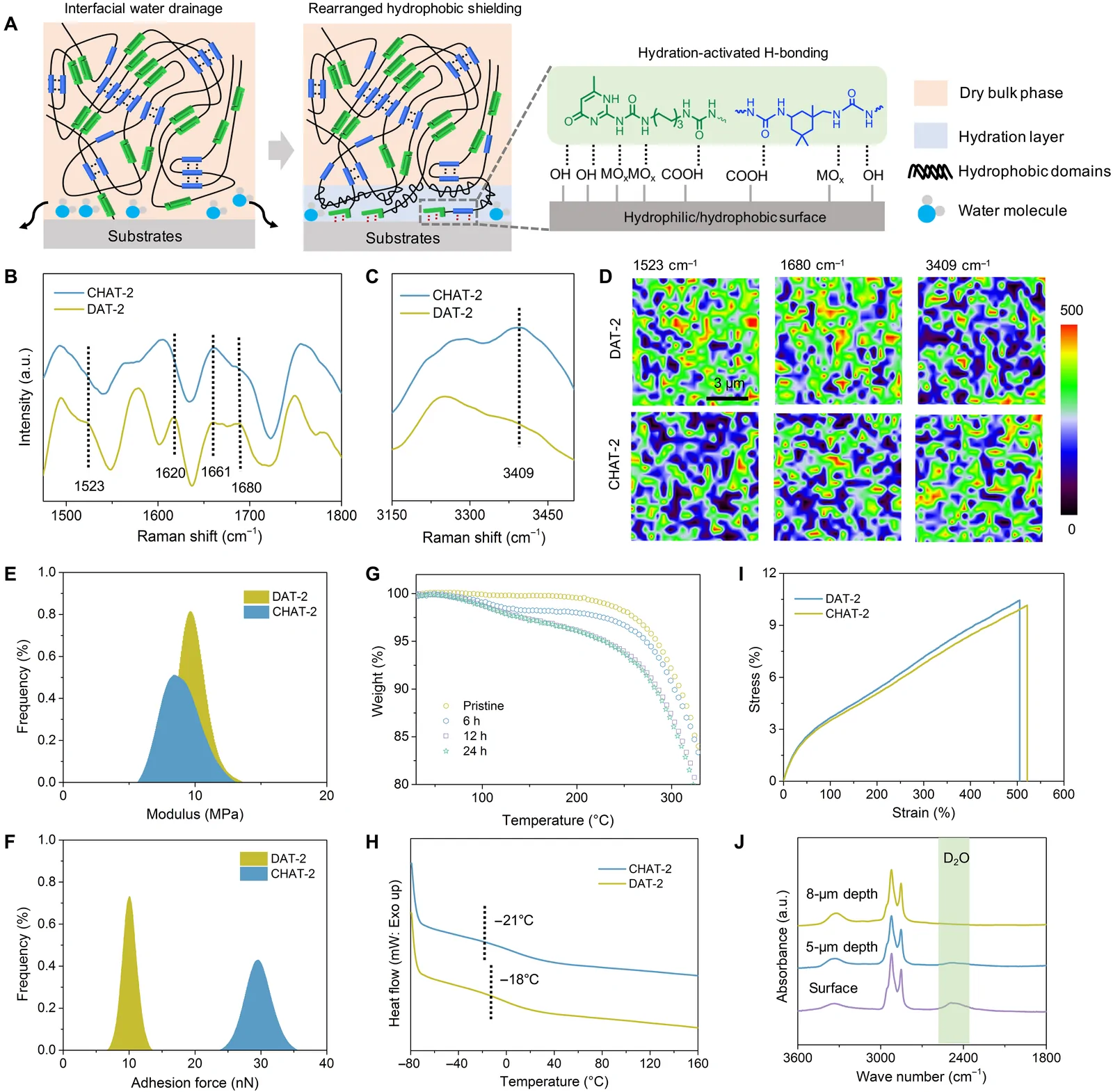
Experimental evidence for confined hydration. (**A**) Schematic illustration of the design principle for spatially confined hydration, which enables strong and tough underwater adhesion. This is achieved by (i) hydrophobic surface domains that actively exclude bulk water; (ii) a hydrated interfacial layer where plasticized hydrogen bonds enhance conformal contact; and (iii) a nonhydrated bulk phase that preserves mechanical integrity. (**B** to **D**) Raman spectra and mapping reveals hydration-induced changes in UPy dimer dissociation (1661 versus 1680 cm^−1^) and water penetration (3409 cm^−1^). (**E** to **F**) AFM nanomechanical profiles shows modulus and adhesion force distributions of DAT-2 and CHAT-2 surfaces. (**G**) TGA curves with varying immersion time in water. [(h) hours] (**H**) DSC profiles of DAT-2 and CHAT-2. (**I**) Tensile stress–strain curves of DAT-2 and CHAT-2. (**J**) The absorbance of D_2_O at various penetration depths analyzed by micro-ATR-FTIR. Scale bars, 3 μm (D).

Figure 3A schematically illustrates CHAT’s adhesion mechanism in wet environments. The adhesive’s surface layer, composed of flexible hydrophobic polyetheramine chains, actively displaces interfacial water molecules upon contact with the substrate. Limited water absorption into this interfacial region induces a controlled plasticization effect, which enhances conformal contact under applied pressure, selectively activates hierarchical hydrogen bonds to create additional interfacial binding sites, and maintains structural integrity through nanoscale reorganization of hydrophobic microdomains. Simultaneously, the nonhydrated bulk phase preserves its mechanical robustness via strong, ordered hydrogen-bonded nanostacks (providing structural stability) and energy-dissipating weak hydrogen-bonded phase (enabling high toughness). Therefore, this spatially confined hydration creates an optimal balance between surface adaptability (for bonding), bulk cohesion (for load-bearing), and energy dissipation (for crack resistance).

To investigate how bulk mechanical properties influence adhesion performance, we characterized the tensile behavior of hydrated CHATs (prepared by 24-hour immersion of DATs followed by surface drying). CHAT-2 demonstrated optimal mechanical properties: a tensile strength >10 MPa, a balanced Young’s modulus of 9.9 ± 1.7 MPa, and a maximum toughness of 31.2 ± 2.4 MJ/m^3^ (figs. S19 and S20 and table S4). These metrics create a critical synergy of bulk cohesion and interfacial adhesion, which facilitates interfacial adaptability and energy dissipation.

To explore the effect of interfacial hydration on surface contact, we performed water contact angle (WCA) measurements and scanning electron microscope (SEM) analysis. The surface of DAT-2 showed a WCA of 92.1° ± 1.9°, higher than that of CHAT-2 (67.3° ± 1.6°) (fig. S21), which indicates that surface hydration enables rearrangement of polar groups and reduces WCA marginally, but hydrophobicity persists. This surface hydration promotes intimate contact and minimizes interfacial defects between CHAT-2 and the substrate, as verified by the cross-sectional SEM image (fig. S22).

To investigate how interfacial hydration governs surface hydrogen-bond chemistry and microstructure, we combined Raman spectroscopy and atomic force microscopy (AFM). Raman mapping of CHAT-2’s hydrated layer (Fig. 3, B to D) validated hydrogen bond reorganization and hydration activation. Upon water exposure, a corresponding increase in the intensity at 1661 cm^−1^ (dissociated UPy motifs) was accompanied by a decrease at 1680 cm^−1^ (associated UPy dimers). Further evidence for the disruption of hydrogen bonding upon hydration comes from the enhanced water signal at 3409 cm^−1^, alongside reduced peaks for N-H vibration (1523 cm^−1^) and ordered urea (1620 cm^−1^). These collective changes confirm the hydration-triggered dissociation of the polar hydrogen-bond networks. Surface modulus and adhesion force measurements revealed controlled softening and adhesion enhancement. As shown in AFM-based indentation (Fig. 3, E and F), CHAT-2’s modulus was slightly reduced compared to DAT-2, while the adhesion force of CHAT-2 showed a threefold increase from 10.1 ± 2.9 nN to 29.5 ± 5.7 nN. This hydration-programmed interface achieves dual optimization: (i) surface plasticity (water-mediated H-bond dissociation enables substrate conformability); (ii) binding capacity (reconstructed H-bond networks enhance interfacial interactions). The mechanistic insights directly correlate with CHAT-2’s macroscopic performance—its record lap shear strength (7 MPa) and interfacial toughness (6 kJ m^−2^) (figs. S9 and S15).

Our systematic characterization reveals that CHAT-2’s unique hydration behavior is confined to a thin surface layer while preserving bulk integrity. Thermogravimetric analysis (TGA) measurements revealed rapid water uptake reaching saturation at 2.5 wt % within 12-hour immersion, without further substantial absorption after 24 hours (Fig. 3G). This hydration kinetics directly correlated with the interfacial toughness plateau (fig. S11A), confirming that both interfacial adhesion and bulk hydration stabilize concurrently after 12 hours. DSC analysis (Fig. 3H) indicated a slight reduction in glass transition temperature for CHAT-2 compared to DAT-2. Mechanical tensile characterization (Fig. 3I and fig. S23) demonstrated that CHAT-2 maintained comparable Young’s modulus and toughness to DAT-2 despite hydration. This invariance in bulk mechanical and thermal properties suggests that hydration is localized to a thin surface layer, with minimal penetration into the tape’s interior. To determine the hydration depth, CHAT-2 was immersed in D_2_O for 24 hours and then cross-sectioned. The chemical composition gradient was subsequently measured as a function of depth. Micro–attenuated total reflection (ATR)–FTIR profiles of CHAT-2 (Fig. 3J) revealed strong deuteration signals at the surface with a sharp decay at 5-μm depth and negligible penetration beyond 8 μm. Similarly, SAXS profiles (fig. S24) of CHAT-2 maintained identical scattering patterns in 8-μm sections with the pristine DAT-2 sample. Raman spectra (fig. S25) confirmed that the intensities of UPy motifs at 8-μm depth returned to those of the dry state. This combination of experimental characterizations confirms the confined hydration to superficial layers and bulk mechanical integrity preserved.

Therefore, this spatially confined hydration mechanism enables two critical functions: (i) surface-specific water plasticization and chemical reorganization to enhance conformal contact and interfacial bonding, while (ii) preserving the structural integrity and mechanical properties of the bulk material. The sharp hydration gradient arises from the material’s engineered hydrophobic nanodomains that restrict water penetration beyond the superficial layers. CHAT-2’s spatial hydration management creates a “best of both worlds” scenario: surface hydrated enough for adhesion (polar groups activated) and bulk hydrophobic enough to resist water infiltration (mechanical integrity preserved).

### Simulation evidence for confined hydration

**Fig. 4.**
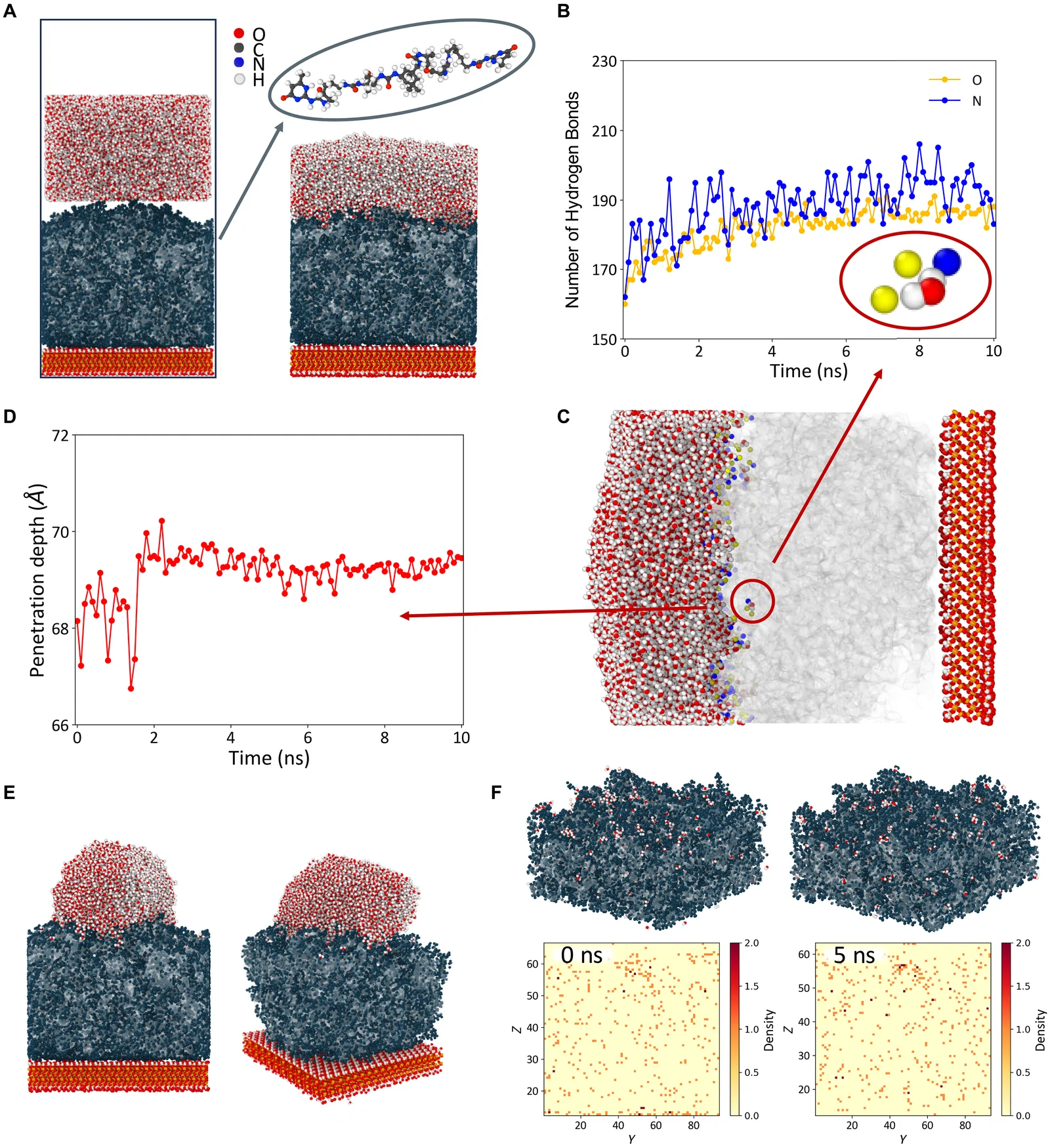
MD simulation. (**A**) The initial and final stable configurations of a three-layer model, which includes 10,000 water molecules, after a 10-ns simulation. (**B**) The number of hydrogen bonds at the polymer interface, within a proximity of 3.5 Å to water molecules. (**C**) The distributions of nitrogen and oxygen atoms at the water-polymer interface, which are highlighted in blue and yellow. (**D**) The maximum penetration depth of water molecules within the polymer surface layer. (**E**) The formation of a discrete, periodic water column with 6000 water molecules in the top layer. (**F**) The initial and final snapshots (after 5-ns simulation at 298 K) along with the corresponding density projection map of water.

Molecular dynamics (MD) simulations were conducted to investigate water penetration into the polymer adhesive and its interaction with the underlying silica substrate. Our simulations reveal that water molecules are thermodynamically driven to the polymer surface, with negligible infiltration into the bulk or migration to the polymer-silica interface. Figure 4A shows the initial and equilibrium configurations of a three-layer system (10,000 water molecules) after 10 ns of simulation. Water molecules rapidly organize into a surface hydration layer, with no visible penetration into the polymer bulk. Figure 4C highlights the final distribution of interfacial water molecules, with polar atoms (N/O, colored blue and yellow) anchoring water clusters. This spatial restriction aligns with the maximum penetration depth tracked in Fig. 4D, where the deepest water molecule remains within 10 Å of the surface. The number of hydrogen bonds (N/O–water pairs within 3.5 Å) plateaus after 5 ns (Fig. 4B), confirming that hydration is limited to the interface. To assess the wettability, we reduced the water layer to 6000 molecules (Fig. 4E). The formation of discrete water columns (contact angle ~90°) suggests dominant macroscopic hydrophobicity despite nanoscale hydration at polar sites. This aligns with the dispersion model (Fig. 4F). When water is forcibly dispersed into the polymer, molecules migrate to N/O groups but fail to aggregate or penetrate further, underscoring the polymer’s kinetic barrier to bulk diffusion.

The MD simulations provide mechanistic insights into water confinement: The hydrophobic polymer backbone acts as a kinetic barrier, suppressing bulk diffusion, while polar functional groups (N/O) thermodynamically stabilize water at the interface through hydrogen bonding, forming a nanoscale hydration layer. This dual mechanism, combining thermodynamic preference for interfacial hydration with kinetic resistance to bulk permeation, is further corroborated by experimental observations (Fig. 3), demonstrating that water is persistently confined to the polymer surface. Although MD simulations operate on shorter timescales than experimental processes, the system reaches quasi-equilibrium within nanoseconds, as evidenced by stabilized interfacial hydrogen bonding and penetration depth profiles. The absence of polymer structural relaxation and persistent surface localization suggest that these conclusions would remain valid over longer timescales. These findings establish a clear design strategy for water-resistant adhesives: optimize the hydrophobic matrix to resist bulk penetration while strategically incorporating polar moieties to control interfacial hydration behaviour.

## DISCUSSION

In summary, we demonstrate how spatially confined hydration—enabled by molecularly engineered SCNs—achieves an unprecedented balance between interfacial adhesion and bulk cohesion in underwater adhesive tapes. The molecular design—combining robust UPy dimers, energy-dissipating IPDI spacers, and flexible hydrophobic polyetheramine chains—enables nanoscale hydration localization at the interface while maintaining bulk integrity. CHAT-2 achieves both remarkable interfacial toughness (>6 kJ m^−2^ on metals; >1 kJ m^−2^ on low-energy surfaces) and high shear strength (>7 MPa on high-energy surfaces; >1.5 MPa on low-energy surfaces). CHAT-2’s superior fatigue resistance arises from hierarchical and balanced molecular architectures, hydration-activated energy dissipation, and cohesion-adhesion synergy. Moreover, CHATs demonstrate exceptional resistance to extreme conditions and long-term stability in saline environments. These capabilities address critical limitations of conventional underwater adhesives, positioning CHATs as a game-changing solution for the most demanding marine applications in offshore infrastructure repair, deep-sea exploration, and marine energy systems. By reimagining interfacial water as a programmable design element rather than a failure point, this work establishes a paradigm in wet adhesion—one that synergizes molecular-scale engineering with precision hydration control at the mesoscale. This fundamental advance opens possibilities for next-generation marine technologies.

## MATERIALS AND METHODS

### Materials

2-Amino-4-hydroxy-6-methylpyrimidine (99%) was purchased from Acros Organics. IPDI, polypropylene glycol diamine (M_n_ = ~2000 g mol^−1^), and the rest of the chemicals and solvents were purchased from Sigma-Aldrich and used as received. All the commercial double-sided tapes including 3M VHB 4910, 3M VHB 5925, 3M VHB 4950, 3M 55230 EVA, Gorilla, and Tesa were used as purchased.

### Synthesis of 2-(6-isocyanato-hexylamino)-6-methyl-4[1H]-pyrimidinone

2-(6-Isocyanato-hexylamino)-6-methyl-4[1H]-pyrimidinone (UPy-NCO) was prepared as reported (*30*, *48*). 2-Amino-4-hydroxy-6-methylpyrimidine (10 g, 79.9 mmol) was added to a 250-ml round bottomed flask. Hexamethylene diisocyanate (HMDI, 100 ml, 624 mmol) and pyridine (7 ml) were then added, the flask was fitted with a reflux condenser, and the mixture was stirred at 100°C overnight under dry nitrogen. Pentane (30 ml) was then added, and the solid product was collected by filtration. The white powder was washed three times with 125-ml portions of acetone to remove unreacted HMDI and then dried overnight under high vacuum at 60°C.

### Synthesis of SCNs

UPy-NCO (0.584 g, 2 mmol), IPDI with different molar ratios (table S1), and chloroform (40 ml) were charged into a 250-ml three-neck flask fitted with a reflux cooler. After mixing well under 300 rpm mechanical agitation, polypropylene glycol diamine with corresponding quantity dissolved in chloroform (10 ml) was added dropwise. After 6 hours of reaction at 70°C under nitrogen protection, the solvent was removed, and the solid product was redissolved in 20 ml of chloroform and then precipitated in 60 ml of acetone with three times. The product was subsequently dried overnight at 60°C under a vacuum condition.

### Preparation of self-standing DAT and CHAT samples

The resultant SCNs were hot-pressed to yield corresponding films on a glass slide after cooling to room temperature. The thickness of the DAT samples was fixed to be 50 μm unless otherwise mentioned. CHAT samples were prepared by soaking the corresponding DAT sample in water for 24 hours immediately before testing.

### Characterization

^1^H (400 MHz) NMR spectra were recorded on a Bruker DRX 400 spectrometer in chloroform-d. GPC was conducted using an Agilent Technologies 1260 Infinity, and the data were processed using Agilent GPC/SEC software; polystyrene was used as the calibrant. Samples for GPC analysis were dissolved in analytical grade THF (2 mg/ml). FTIR spectra were obtained using a PE Spectrum 100 between 4000 and 600 cm^−1^ with a resolution of 4 cm^−1^ and 10 scans per data point. The peak of D_2_O measured at different depths was validated by micro-ATR-FTIR. The germanium crystal as a single-reflection ATR accessory was used, with a refractive index of 4. The process was conducted within 5 min to reduce the evaporation of hydrated water in the surface layer. The TGA was conducted with 10-mg samples in N_2_ at temperatures ranging from 30° to 600°C with a heating rate of 10°C min^−1^. DSC measurements were performed in N_2_ using an MDSC 2910 system, which is operated in the range −80 to 160°C at a heating/cooling rate of 10°C min^−1^, and the sample was 5 mg. Unless otherwise stated, data from the second heating cycle and the reverse heat flow curve are presented. XRD analysis (Bruker AXS, D2 PHASER) was conducted with Cu Kα radiation (λ = 0.15418 nm) at 30 kV to analyze the semicrystalline structures. AFM measurements were carried out using Si_3_N_4_ tips (DNP-B, Bruker) in the PeakForce quantitative nanomechanical property mapping mode on a bioscope catalytic AFM (Bruker Dimension Icon with ScanAsyst) (*33*, *49*, *50*). Under ambient circumstances, contact angle measurements were made with OCA 20 equipment (Data Physics, Germany). Each set of data was based on the results of at least 10 separate experiments. The cross-sectional image of the interface between CHAT-2 and the glass substrate was characterized by an environmental SEM (FEI Quanta 600) with an accelerating voltage of 5 kV. The Raman measurements were conducted with an alpha 300R WITec confocal Raman system (WITec GmbH, Ulm, Germany) (*51*). The excitation laser at 532 nm was maintained at a low power output of 10 mW. The Raman mapping of the scan sizes was 10 μm by 10 μm.

### Determination of domain spacing and radius of gyration

SAXS measurements on the adhesive tape were conducted using SAXSpace equipped with a microfocus x-ray source operating at λ = 0.15418 nm to analyze segregated microdomains (*30*, *52*). The distance between sample and the detector is 1060 mm, and the data collecting duration is 600 s. The SAXStreat and SAXSquant software supplied by the vendor (Anton Paar) were used to integrate two-dimensional images azimuthally, adjust for background scattering and normalize them. The resulting scattering intensity *I(Q)* was displayed as a function of scattering vector *Q*. The radius of gyration (*R_g_*) can be obtained from the Guinier profile (*1*, *29*)

$$I\left(q\right)=a_{0}\text{exp}\left(-\frac{R_{g}^{2}}{3}q^{2}\right)$$ (1)

where *q* is scattering vector, *I(q)* is the scattering intensity, and *a_0_* is a constant.

Take the logarithm of equation

$$\text{ln}\left[I\left(q\right)\right]=\text{ln}a_{0}-\frac{R_{g}^{2}}{3}q^{2}$$ (2)

### Tensile measurements

The DAT samples were tested as prepared, while CHAT samples were prepared by soaking the corresponding DAT tapes in water for 24 hours immediately before tensile testing. The tensile machine (50-N load cell; INSTRON-5566) with the ASTM D2256 standard was adopted with a constant tensile speed of 50 mm min^−1^. The sample size of 30-mm length and 20-mm width was used with a gauge length of 10 mm. The tests were repeated three times in total at room temperature with the average values recorded. The tensile strength was determined by dividing the maximum force by the adhesion area. The Young’s modulus was calculated from the starting slope of the stress-strain curves. The tensile toughness was calculated by the area beneath the stress-strain curve.

### Adhesive measurements

Before adhesion, substrates including copper, steel, aluminum, glass, epoxy, PMMA, wood, and PTFE, were cleaned with acetone, ethanol, and distilled water. To prepare the bonded joints, the DAT or CHAT samples were immersed in water for 0 or 24 hours. Subsequently, they were pressed against the specific substrates under a preload of 10 kPa for 20 s.

To measure interfacial toughness, the bonded samples with widths of 20 mm were prepared and tested by a 90° peeling test (ASTM D2861) using a mechanical testing machine (50-N load cell; INSTRON-5566). All tests were fully submerged in a water bath and conducted with a constant peeling speed of 50 mm min^−1^. Seventy-micrometer-thick Kapton films were used as the stiff backing of the tapes. The interfacial toughness was calculated by dividing the average peeling force at the plateau by the width of the tested adhesive tape.

To measure lap shear strength, the bonded samples with an adhesion area of width 20 mm and length 10 mm and a thickness of 50 μm were prepared and tested by a standard lap-shear test (ASTM 2256) with a mechanical testing machine (50-N load cell; INSTRON-5566). All tests were conducted fully submerged in a water bath with a constant peeling speed of 50 mm min^−1^.

To evaluate the adhesion stability under harsh conditions, DAT samples were immersed in PBS (pH 7), urea (5 mg/ml), acidic (pH 1), basic (pH 13), and seawater (approximately 3.5 wt % NaCl) for 24 hours, respectively. Subsequently, they were pressed against a glass substrate under a preload of 10 kPa for 20 s. The shear strength was determined by dividing the maximum force by the adhesion area. The tests were repeated three times in total with the average values recorded.

### Interfacial fatigue threshold measurements

To determine the interfacial fatigue threshold, 90° peeling tests were further conducted with the CHAT samples fully submerged in a water bath. This ensures that the hydrated interface remains in its functional state throughout the entire mechanical test. The tests were run for a sufficient number of cycles to accurately measure the crack growth rate, *dc/dN*, at each applied energy release rate (*G*). The number of cycles per *G* level ranged from 1000 to 30,000 cycles, depending on the crack propagation rate. At higher *G* values, fewer cycles were needed. Near the fatigue threshold (low *G* values), tests were run for up to 30,000 cycles to confirm that the crack growth rate was negligible. A frequency of 0.5 Hz (a period of 2 s per cycle with 1-s loading and 1-s unloading) was used for all cyclic peeling tests. The crack length (*c*) was monitored as a function of cycle number (*N*) for each fixed G. The crack growth rate, *dc/dN*, was then calculated from the slope of the linear region of the *c* versus *N* curve. A plot of *dc/dN* versus *G* was constructed, and the interfacial fatigue threshold (Γ_0_) was determined by linearly extrapolating this plot to the *G* axis where *dc/dN* = 0.

### MD simulations

To assess the hydration behavior at the polymer-water interface and the spatial confinement of water molecules to the polymer matrix, we constructed three MD models to simulate the interaction between water molecules and the target polymer adhered to a silica substrate. The models consisted of two three-layer models and one dispersion model, each designed to provide insights into the polymer’s hydration properties. The three-layer models were composed of a water layer, a target polymer layer, and a silica substrate, stacked in that order. A 150-Å vacuum buffer layer was positioned above the water layer to prevent interactions between the water and the silica substrate due to periodic boundary conditions. The polymer in the MD simulation is represented by its smallest repeating monomer unit due to the size limitations of the simulation system, as illustrated in Fig. 4A. The polymer layer in three-layer models contains 300 polymer monomers. The silica substrate beneath the polymer has a periodic structure in the *xy* plane and is saturated with hydroxyl groups on both its upper and lower surfaces. The two three-layer models differ in the number of water molecules in the water layer above the polymer. One model includes 10,000 water molecules, forming a continuous and periodic water film in the *xy* direction. In contrast, the other model has 6000 water molecules, insufficient to form a continuous water film, thereby allowing the observation of water cohesion on the polymer surface and the wettability of the polymer surface. The structural framework of these models is shown in Fig. 4. The water dispersion model, which included only the polymer and water molecules, featured 412 water molecules randomly distributed within a polymer layer composed of 200 target polymer molecules, providing a scenario to study the confinement of water molecules within the polymer matrix.

### Force-field parameters and simulation settings

All MD simulations were performed using the LAMMPS (Large-scale Atomic/Molecular Massively Parallel Simulator) package (*53*). The interactions within the polymer molecules were described using the all-atom optimized potentials for liquid simulations (OPLS-AA) force-field parameters (*54*), while the interaction parameters for the silica surface atoms were derived from the Clay force field (*55*). Cross-interactions between different atom types were calculated using the arithmetic mixing rule. In the simulations, all atoms on the silica surface, except only for the oxygen and hydrogen atoms of the hydroxyl groups, were treated as rigid bodies. Before the simulations, energy minimization of all models was performed using the steepest descent method, and the resulting configurations were used for MD simulations. The simulations investigated the spreading and penetration behavior of water droplets on the polymer surface under the NVT ensemble. The system temperature was maintained at 298 K using a Nose-Hoover thermostat with a coupling constant of 0.1 ps (*56*, *57*). Time steps varied depending on the model to ensure accurate interaction calculations. For the three-layer models, a time step of 0.2 fs was used, while for the water dispersion model, a time step of 0.5 fs was used. These shorter time steps allowed for more precise simulation of molecular interactions. Periodic boundary conditions were applied in all three directions throughout the simulations, and each simulation was run for up to 5 or 10 ns, depending on the model, to ensure sufficient time for the water droplets to spread and penetrate the polymer layer.
