# Supplementary material for: Spatially confined hydration for robust underwater adhesion
Source: Sci Adv. 2025 Nov 5;11(45):eaea3097. doi: 10.1126/sciadv.aea3097 (PMC12588289; doi:10.1126/sciadv.aea3097)
Supplement: Supplementary file 1 — Figs. S1 to S25 Tables S1 to S4 [file sciadv.aea3097_sm.pdf]

Science Advances
AAAS

Supplementary Materials for

Spatially confined hydration for robust underwater adhesion

Gang Lu *et al.*

Corresponding author: Gang Lu, ganglu7@seas.upenn.edu; Jian Lu, jlu@yic.ac.cn

Sci. Adv. **11**, eaea3097 (2025)
DOI: 10.1126/sciadv.aea3097

This PDF file includes:

Figs. S1 to S25
Tables S1 to S4

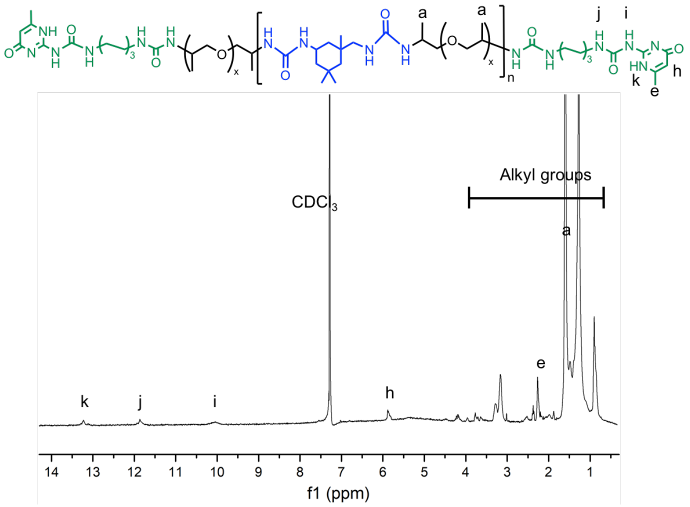

Fig. S1. ^{1}H NMR spectrum of SCN-2 in $CDCl_3$. The resonances between 10.0 and 14.0 ppm are characteristic of the N−H protons in the strongly hydrogen-bonded UPy dimers.

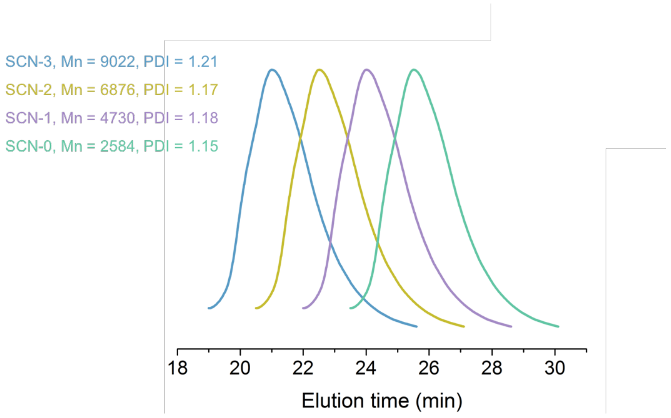

Fig. S2. GPC profiles of the resultant oligomers.

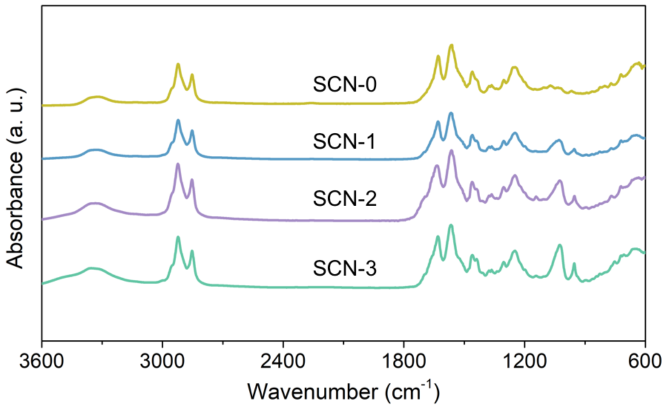

Fig. S3. FTIR spectra of the resultant oligomers.

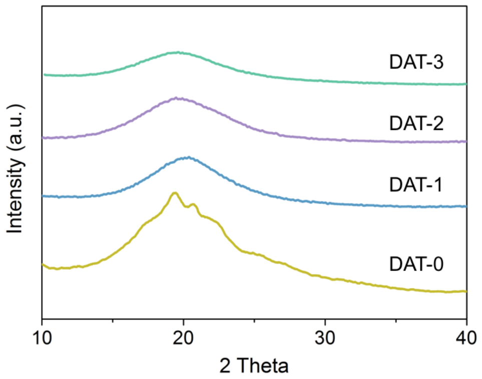

Fig. S4. X-ray diffraction profiles of DAT 0-3. The sharp diffraction peaks for DAT-0 indicate semi-crystallinity from well-defined UPy nanostacks. The decreasing peak intensity from DAT-1 to DAT-3 demonstrates how increasing the flexible polyetheramine content disrupts crystalline order, resulting in more amorphous materials.

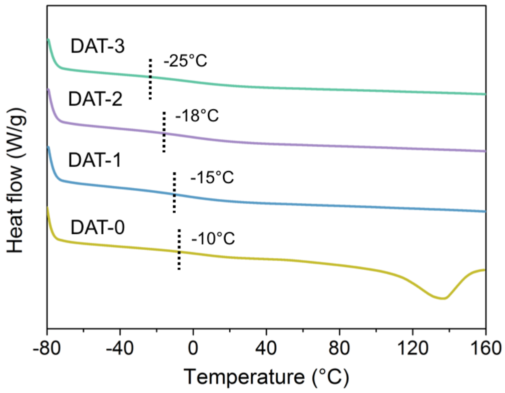

Fig. S5. DSC profiles of the resultant DAT 0-3.

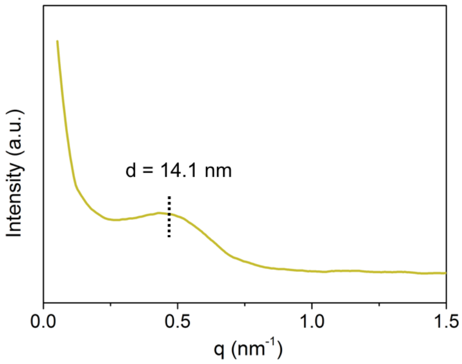

Fig. S6. SAXS analysis of DAT-2.

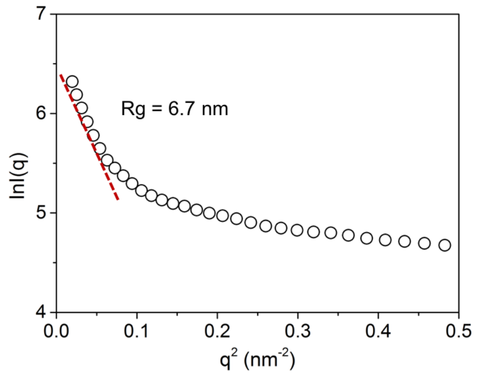

Fig. S7. The radius of gyration of DAT-2.

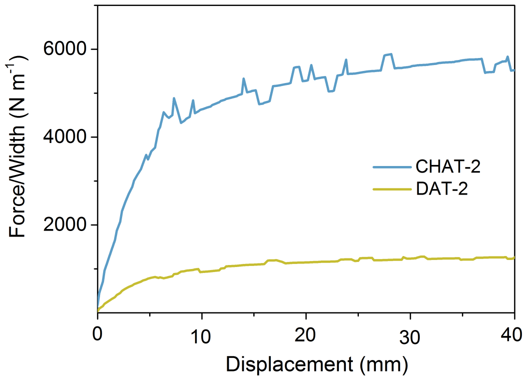

Fig. S8. The peeling testing curves of DAT-2 and CHAT-2.

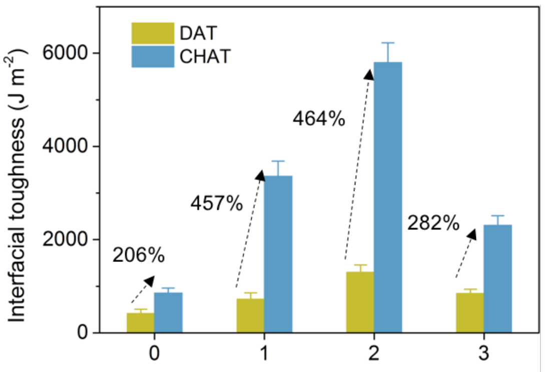

Fig. S9. Comparison of interfacial toughness of DAT and CHAT samples tested on glass substrates. Data are means ± SD, n = 3.

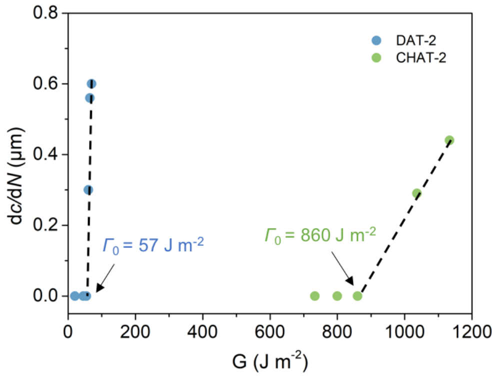

Fig. S10. Interfacial fatigue threshold measurements of DAT-2 and CHAT-2 on the glass substrate.

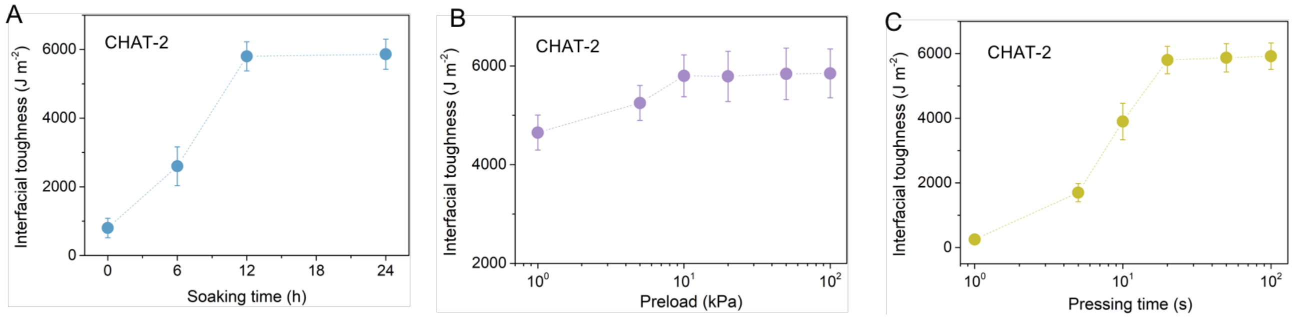

Fig. S11. The impact of soaking time, preload, and pressing time on interfacial toughness of CHAT-2. (A) Interfacial toughness versus immersion time for CHAT-2 on glass substrates, with a preload of 10 kPa for 20 seconds. (B) Interfacial toughness versus preload for CHAT-2 on glass substrates, with 24 hour-soaking time and a pressing time of 20 seconds. (C) Interfacial toughness versus pressing time for CHAT-2 on the glass substrates, with 24 hour-soaking time and a preload of 10 kPa. Data in (A), (B), and (C) are means ± SD, n = 3.

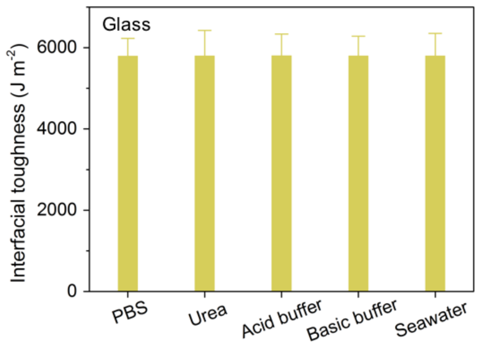

Fig. S12. Interfacial toughness of CHAT-2 samples that were prepared by immersion in various aqueous solutions including PBS (pH 7), urea (5 mg/mL), acidic (pH 1), basic (pH 13), and saline (3.5 wt% NaCl) for 24 hours, followed by a preload of 10 kPa for 20 seconds. Data are means ± SD, n = 3.

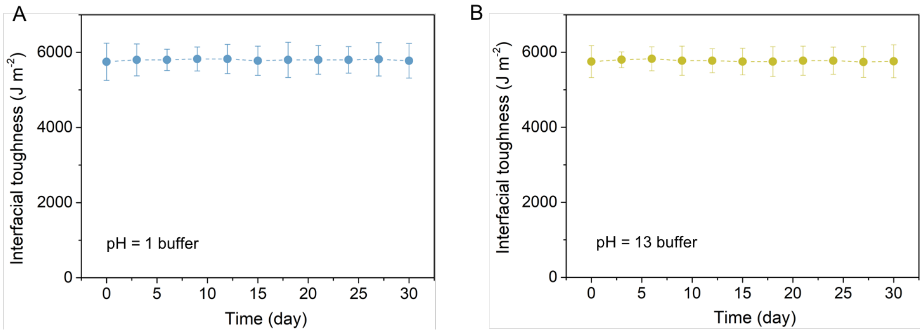

Fig. S13. The interfacial toughness of CHAT-2 bonded on the glass surface with 30-day immersion in acidic or alkaline buffer, tested at various time points. Data in (A) and (B) are means ± SD, n = 3.

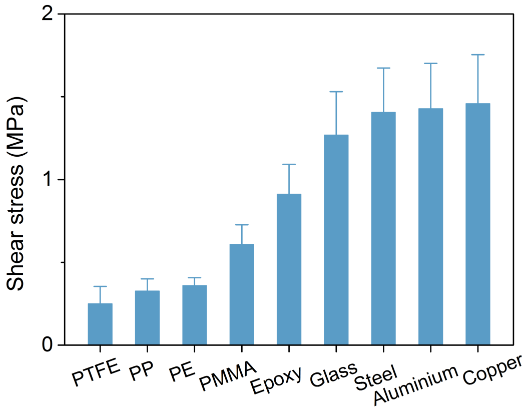

Fig. S14. The shear strength of DAT-2 tested on diverse substrates. Data are means ± SD, n = 3.

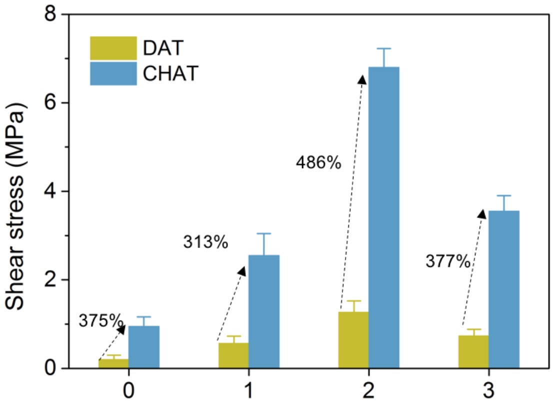

Fig. S15. Shear strength of DAT-2 and CHAT-2 tested on the glass substrate. Data are means ± SD, n = 3.

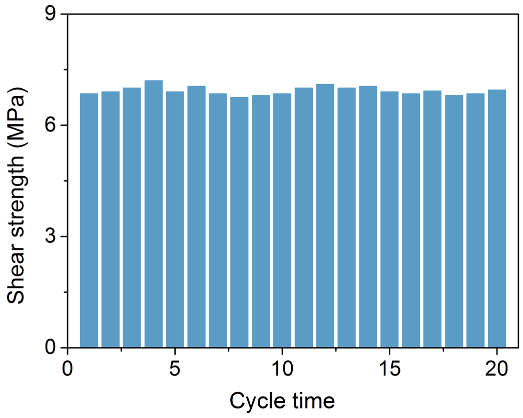

Fig. S16. Shear strength of CHAT-2 tested on glass substrates with 20-cycle debonding and bonding.

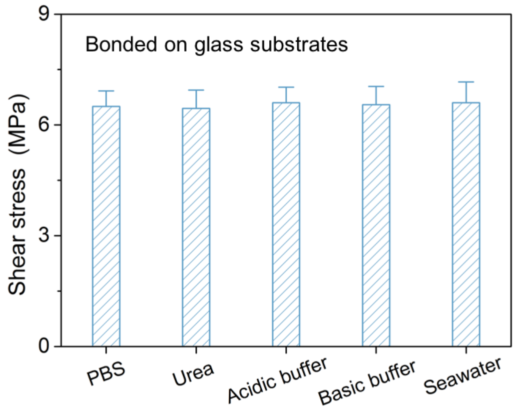

Fig. S17. Shear stress measurements of CHAT-2 samples that were prepared by 24-h immersion in diverse aqueous solution followed by a standard bonding process (a preload of 10 kPa for 20 seconds). Data are means ± SD, n = 3.

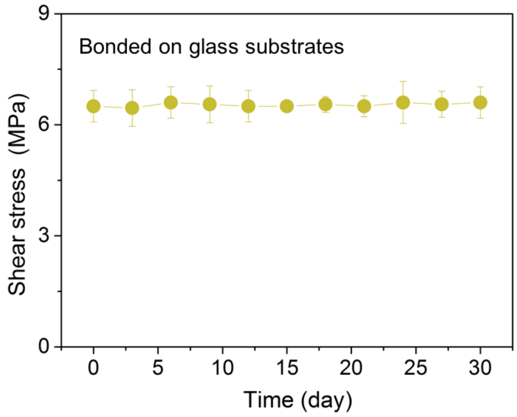

Fig. S18. Shear stress measurements of CHAT-2 that was immersed in saline water for 30 days and tested at specific immersion time. Data are means ± SD, n = 3.

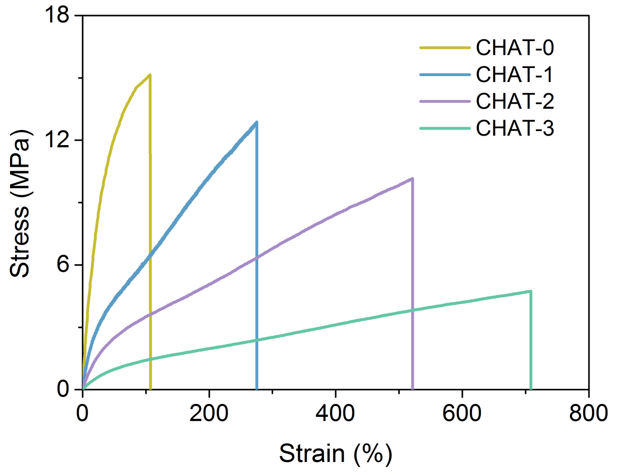

Fig. S19. Tensile measurements of CHAT samples.

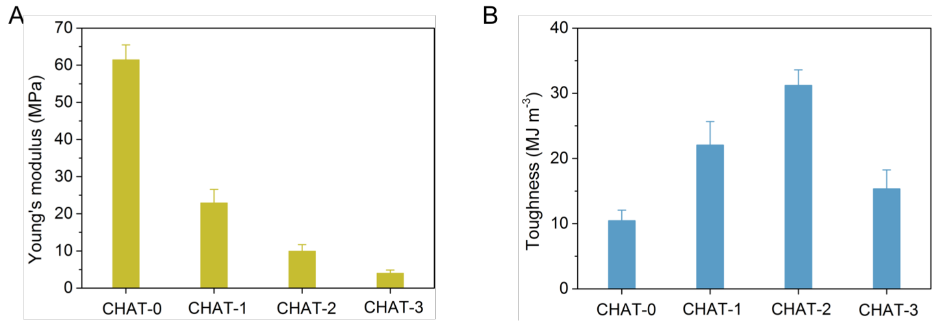

Fig. S20. Young's modulus and toughness of CHAT samples, calculated from Fig. S19. Data in (A) and (B) are means ± SD, n = 3.

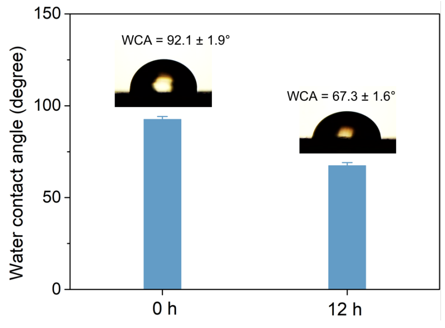

Fig. S21. Water contact angle measurements on the surface with varying water soaking time. Data are means ± SD, n = 10.

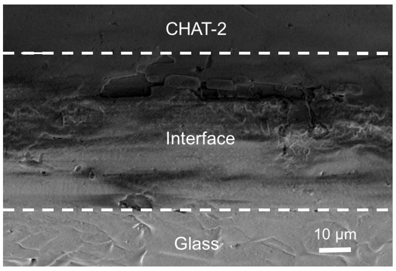

Fig. S22. The cross-sectional SEM image showing the interface between CHAT-2 and the glass substrate.

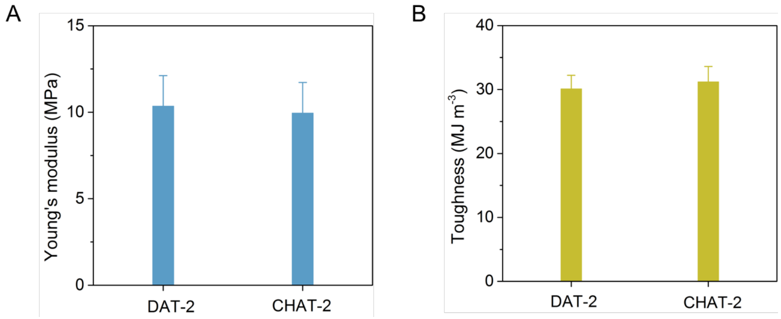

Fig. S23. Young's modulus and toughness of DAT-2 and CHAT-2, calculated from Fig. 3I. Data in (A) and (B) are means ± SD, n = 3.

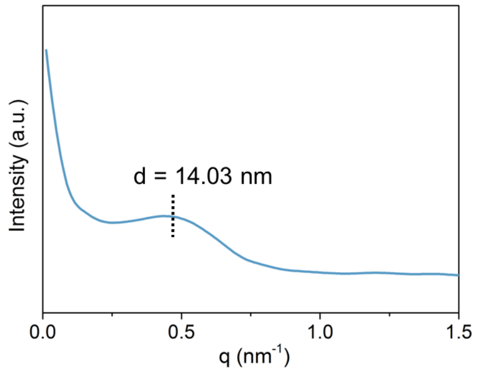

Fig. S24. SAXS analysis on the internal structure of CHAT-2.

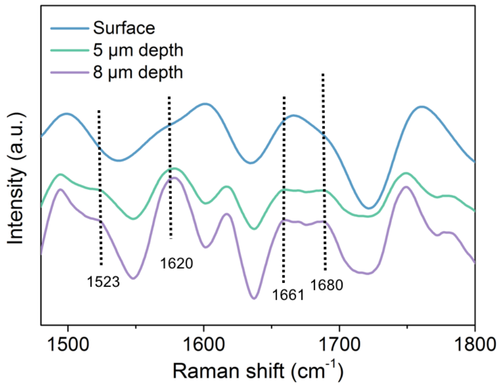

Fig. S25. Raman spectra of CHAT-2 at different depth.

Table S1. The feed ratio of functional motifs for the preparation of supramolecular cooperative networks.

Compound (mmol)	DAT-0	DAT-1	DAT-2	DAT-3
UPy-NCO	2	2	2	2
OPG-2NH_2	1	2	3	4
IPDI	0	1	2	3

Table S2. Benchmarking with the literature-reported and commercial adhesive tapes in interfacial toughness.

Materials	Interfacial toughness (kJ m^{-2})	Substrate type	Source	Literature
CHAT-2	5.8	Glass	Our work	
	6.21	Steel		
	6.24	Copper		
	3.1	Epoxy		
	1.59	PMMA		
	1.12	PE		
	0.98	PP		
	0.91	PTFE		
PDMA	0.03		ACS Appl. Mater. Interfaces 2019, 11, 24802	36
PAAm	0.17		Adv. Funct. Mater. 2020, 30, 2003207	37
NaPAA	0.45		ACS Appl. Mater. Interfaces 2019, 11, 24802	36
AUA	0.458	PET	Nat. Commun. 2023, 14, 6563	22
PAA-PAAm	0.35	Glass	Adv. Funct. Mater. 2020, 2003207	37
PS-PAAm	1		Adv. Sci. 2022, 9, 2105742	38
PU-PVP	1.13	Solid substrates	Adv. Mater. 2021, 2007301	28
P(BA-co-IBA)0.4	2.05	Glass	Adv. Sci. 2022, 9, 2105742	38
Chitosan/Alg/PAAm	2.4		Adv. Mater. 2019, 31, 1806948	24
Alg/PAAm	2.7	Steel	ACS Appl. Mater. Interfaces 2019, 11, 24802	36
Chitosan	3.1		PNAS, 2024, 121, e2304643121	39
CED hydrogel	3.3	Glass	Adv. Funct. Mater. 2023, 2306914	29
3M VHB 4910	2.5	Glass	Commercial	
3M VHB 5925	3	Glass		
3M VHB 4950	4.4	Glass		
Gorilla	3.2	Glass		
Tesa	1.6	Glass		

Table S3. Benchmarking with the literature-reported and commercial adhesive tapes in lap shear stress.

Materials	Shear stress (MPa)	Substrate type	Source	Literature
CHAT-2	1.7	PTFE	Our work	
	2.2	PP		
	2.6	PE		
	3.5	PMMA		
	4.7	Epoxy		
	5.3	Wood		
	6.5	Glass		
	7.3	Steel		
	7.4	Aluminium		
	7.5	Copper		
Self-hydrophobization hydrogel	0.035	PP	Adv. Funct. Mater. 2020, 30, 1907064	40
Phase Change Hydrogels	0.945	Wood	Adv. Funct. Mater. 2023, 33, 2301505	41
Bioinspired adhesives	0.003	Glass	Nat. Commun. 2018, 8, 2218	15
PVHD5	0.74	PS	Adv. Mater. 2024, 2410453	42
PVA/BA dressing	0.61	Glass	PNAS, 2022, 119, e2203074119	43
SLU	1.6	Metal & Plastic	PNAS, 2023, 120, e2301364120	19
TSGT	3.1	Rough surface	Adv. Mater. 2024, 2405511	21
Electro-Ox hydrogel tape	0.68		Nat. Commun. 2021, 12, 7156	27
Double-sided tape	0.12	Tissue	Nature, 2019, 575, 169	20
Fluorine-rich ionogel	5.18	Glass	Mater. Horiz., 2021, 8, 2057-2064	47
Catechol-PVP	1.3		ChemComm.2015,51,9117--9120	44
CD–Azo hydrogels	2.5	Rigid substrates	Angew. Chem. Int. Ed. 2018, 57, 8963–8967	45
Supramolecular velcro	1.12		Angew. Chem. Int. Ed. 2013, 52, 3140 –3144	25
Poly(DMA-MEA)	1.2	Glass	Biomacromolecules 2015, 16, 2265–2275	46
Tesa 4965	1	Glass	Commercial	
3M VHB	0.5	Glass		
3M 9495LE	0.65	Glass		
3M 55230 EVA	0.35	Glass		

Table S4. Mechanical properties of DAT and CHAT samples.

Samples	Tensile strength (MPa)	Strain (%)	Young's modulus (MPa)	Toughness (MJ/m^3)
DAT-0	15.73 ± 1.15	101.22 ± 1.21	64.55 ± 4.74	9.8 ± 1.55
DAT-1	13.02 ± 1.07	259.42 ± 2.55	23.5 ± 1.7	21.45 ± 4.03
DAT-2	10.45 ± 1.07	505.27 ± 2.73	10.35 ± 1.76	30.1 ± 2.12
DAT-3	4.89 ± 0.98	693.66 ± 9.54	4.1 ± 0.8	14.85 ± 2.75
CHAT-0	15.14 ± 0.83	107.34 ± 1.17	61.45 ± 4.04	10.45 ± 1.63
CHAT-1	12.87 ± 1.08	275.39 ± 2.39	22.9 ± 3.67	20.05 ± 3.61
CHAT-2	10.16 ± 0.97	521.37 ± 3.24	9.95 ± 1.76	31.2 ± 2.4
CHAT-3	4.74 ± 0.54	708.25 ± 4.73	4 ± 0.85	15.35 ± 2.9
